# Two Contrasting Patterns and Underlying Genes for Coadaptation of Seed Dormancy and Flowering Time in Rice

**DOI:** 10.1038/s41598-018-34850-5

**Published:** 2018-11-14

**Authors:** Xing-You Gu, Wirat Pipatpongpinyo, Lihua Zhang, Yuliang Zhou, Heng Ye, Jiuhuan Feng

**Affiliations:** 1 Agronomy, Horticulture, and Plant Science Department, South Dakota State University, Brookings, South Dakota 57007 USA; 2 Agricultural College, South China Agricultural University, Guangzhou, 510642 China

## Abstract

Association between seed dormancy (SD) and flowering time (FT) may generate a synergy in plant adaptation. This research aimed to identify patterns and underlying genes of the association in rice (*Oryza sativa*). Four F_2_ and two BC_1_F_1_ populations from crosses of weedy/cultivated rice, and two families of progeny lines from backcrosses were evaluated for variations in time to flowering and germination ability. The two measurements were correlated negatively in the F_2_ and BC_1_F_1_ populations, but positively in advanced generations of the progeny lines. The negative correlations were resulted from linkage disequilibria between SD and FT loci at 7–40 cM apart. The positive correlations arose from co-located SD and FT loci undetectable in the BC_1_F_1_ population. Two independent sets of co-localized loci were isolated as single Mendelian factors, and haplotypes that promote flowering and reduce germination derived from weedy and cultivated rice, respectively. The presence of negative and positive correlations indicates that the rice complex has maintained two contrasting patterns of SD-FT coadaptation, with the positive being “recessive” to the negative pattern. Modeling with isogenic lines suggests that a negative pattern could generate a greater synergy (difference between haplotype variants) than the positive one for seedbank persistence, or enhanced plant adaptation to seasonal changes in temperature or moisture. However, the early-flowering dormant genotype of a positive pattern could also have a selective advantage over its counterpart for weeds to avoid harvesting. The isolated haplotypes could be used to manipulate cultivars simultaneously for germination ability and growth duration.

## Introduction

Seed dormancy (SD) may associate with flowering time (FT) to generate a synergy for plant adaptation to specific ecosystems. The association was inferred by correlations between SD and FT in oats (*Avena spp*.)^1^, rice (*Oryza sativa*)^2^, shepherd’s purse (*Capsella bursa-pastoris*)^3^ and Arabidopsis (*Arabidopsis thaliana*)^4,5^. Such a phenotypic correlation can be partitioned into the genetic and environmental components to identify genes and selective factors that cause simultaneous changes in the associated traits. Furthermore, the genetic correlation can be the overall effect of all pleiotropic or linked genes that express in the same (association) or different (dispersion) directions^6^. With a recent increase in information about quantitative trait loci (QTL) for SD and FT in some species, it is possible to identify the underlying genes and expression patterns and to model the synergy. This knowledge is also important for understanding weed evolution and correlative selection in crop breeding.

Cereal crops are divergent from wild/weed relatives in SD, because domestication tended to select dormancy-reduced variants to promote germination^7^. Thus, hybrid populations from remote crosses, such as weedy/cultivated rice (*O. sativa*), can be used to screen for linkage disequilibria (LD) between genes for SD and its interrelated traits. The conspecific weedy and cultivated rice originated from the wild ancestors *O. rufipogon* and *O. nivara* in the rice complex, and differentiated into tropical and temperate ecotypes, or the *indica* and *japonica* subspecies^8,9^. Similar to the ancestors, tropical ecotypes of weedy rice adapted to the wet/dry climate for flowering in the summer, while temperate ecotypes adapted to the cool fall season for seed set before the winter. A locally adapted weed population usually retained strong SD from the ancestors, but mimicked accompanying cultivars for FT^10^. Genetic analysis for some hybrid populations identified various degrees of negative correlation between germination ability of newly harvested seeds and time to flowering^2,11–14^. The correlations were accounted for by linked SD and FT loci on chromosomes (Chr) 1, 3, 6 or 7. Many more QTL were associated with SD in the rice complex^15–18^. Further research is needed to determine how many SD loci could associate with FT, how two or more LD could be maintained in a species to influence the trait coadaptation, and what is the difference in a synergy or selective advantage between contrasting patterns of SD-FT association in natural or agricultural systems.

In a series of the previous research, we identified 10 QTL for SD (qSD) from a cross of weedy/cultivated rice and introduced the QTL alleles into the cultivar background by generations of recurrent backcrossing and marker-assisted selection^19^. During the introduction process, we discovered associations of *qSD1-1* and 10 with FT in advanced generations of progeny lines. Interestingly, the associations are different in direction from the reported pattern. This discovery prompted us to track the SD-FT association in early generations of hybrid populations from the same and different crosses. Thus, the first objective of this research was to identify patterns of SD-FT association across generations and their underlying genes/QTL. The second objective was to map *qSD1-1* and 10 in a fine scale to delimit the size of haplotypes and estimate their “pleiotropic” effects in an isogenic background. And, the third objective was to discuss implications of the association patterns and underlying genes in plant/weed adaptation or crop improvement.

## Materials and Methods

### Hybrid populations and progeny lines

Data for this research were collected from six hybrid populations in early generations and two independent families of progeny lines in advanced generations. The six (4 F_2_ and 2 BC_1_F_1_) populations were developed for the previous research^20–22^ and used to estimate association patterns of seed dormancy (SD) with flowering time (FT) in diverse backgrounds. Parental lines for each of the six populations are different in SD, but close in FT. The strongly dormant parents were the weed genotypes “Ludao”, “SS18-2” and “TKN12-2” from China, Thailand and Nepal, respectively, and the landrace “N22” from India. The weakly dormant parents were cultivated genotypes from the *indica* (“CO39”, “Dular” and “EM93-1”) or *japonica* (“WYJ”) subspecies.

One family of single-plant-derived progeny lines was used to isolate *qSD1-1* and to track its association with FT across generations. *qSD1-1* was detected and confirmed in BC_4_F_2_ and BC_4_F_3_ populations^23,24^. The population pedigree involved CO39, EM93-1 and SS18-2. CO39 was crossed with SS18-2 to develop an F_2_ population; then an early-maturation F_2_ plant was selected as the non-recurrent parent to cross with the recurrent parent EM93-1 to develop the backcross (BC) populations. The BC_4_F_3_ population was used to estimate the SD-FT correlation and to map the QTL for FT. A BC_4_F_3_ plant was advanced to the BC_4_F_6_ generation by single-plant selection to purify *qSD1-1*’s genetic background and to identify recombinants between markers on the QTL peak-containing interval.

The other family of single-plant-derived progeny lines was used to isolate *qSD10* and to track its association with FT across generations. *qSD10* was detected in a BC_1_F_2_ and confirmed in a BC_1_F_3_ population from the EM93-1//EM93-1/SS18-2 cross^19^. The BC_1_F_2_ population was used to estimate the SD-FT correlation and to map FT QTL. A BC_1_F_3_ plant was advanced to the BC_1_F_7_ generation to purify *qSD10*’s genetic background and to identify recombinants between markers on the QTL peak-containing interval.

### Plant cultivation and trait quantification

To develop segregating populations or progeny lines, seeds from selected plants were air-dried to break the dormancy before germination at 30 °C for 5 days (d). Germinated seeds were transferred to the rice nutrition solution^25^ in 200-well Seed Starting Trays (Bootstrap Farmer). Seedlings were genotyped with selected markers to identify recombinants or to evaluate marker-trait correlations. Genotyped seedlings were transplanted into pots (12 × 12 × 15 cm^3^; one plant/pot), which were filled with a mixture of clay soil and Sunshine medium (Sun Gro Horticulture), in a greenhouse. Day/night temperatures were set at 29°/21 °C and day-lengths were natural, except for winter seasons when supplementary light was applied to maintain a minimum of 12-h light. Plants were tagged for flowering date when the first panicle of a plant emerged from the leaf sheath. Seeds were harvested at 40 d after flowering, air-dried in the greenhouse for 3 d, and stored in a freezer of −20 °C to maintain the status of primary dormancy.

Flowering time was quantified by the period (d) from germination to flowering, and seed dormancy measured by germination percentage of partially after-ripened seeds. The after-ripening treatment was storing seed samples in a temperature-controlled (24–25 °C) lab room for a given period, which was determined by preliminary germination tests. A formal germination test was conducted using three samples of seeds from each plant. A sample of about 50 seeds was distributed in a 9-cm Petri dish lined with a filter paper, and soaked with 8 ml water. Samples prepared for a population or progeny line were placed in an incubator set at 30 °C and dark. Germinated seeds (radicle protrusion >3 mm) were accounted at the 7^th^ d to calculate germination percentage. The mean of three replicates for a plant was used for genetic analysis.

### Marker genotyping and map construction

To genotype selected plants or a segregating population, genomic DNAs were prepared from fresh leaves and the Rice Microsatellites markers^26^ selected to cover a QTL peak-containing region. DNA extraction, marker amplification by polymerase chain reaction (PCR), and PCR product electrophoresis with 6% non-denatured polyacrylamide gel were performed using the previously described methods^21^. Marker genotyping data were used to develop partial linkage maps using MAPMAKER/EXP 3.0^27^. Map distance was converted into centiMorgan (cM) using the Kosambi function^28^ to estimate the strength of a linkage disequilibrium.

### Genetic analysis and QTL mapping

Linear correlation analysis was used to estimate the strength and direction of associations between germination percentage and time to flowering, or between maker genotypes and the measurements. The genotypes were coded as −1 for the EM93-1-like homozygote, 0 for heterozygote, and 1 for the SS18-2-like homozygote to calculate the correlation co-efficient (r).

The composite interval mapping program of Windows QTL Cartographer^29^ was used to map QTL for FT and SD on the partial linkage maps. The program was run at 1-cM walking speed and 1000 permutations at 5% error rate to define QTL peak positions and to estimate QTL additive (*a*) and dominance (*d*) effects or proportion of the phenotypic variance (R^2^) explained by a QTL. The strength of a linkage disequilibrium was estimated by the distance (cM) between the QTL peak positions.

Data from the progeny lines were used to improve the *a* and *d* estimates using the linear regression model:

1 $${{y}}_{j}={\mu }+{\alpha }x+dz+{\varepsilon }_{j}$$

where, *y*_*j*_ is the phenotypic value for plant *j* (*j* = 1, 2 … N); *μ* is the model mean; *x* is the dummy variable for the *a* component taking values −1, 0 and 1 for the three genotypes; *z* is the dummy variable for the *d* component taking values 0.5 for genotype 0, or −0.5 for genotypes −1 or 1; and *ε*_*ik*_ is the error term of the model. Correlation and regression analyses were implemented using SAS program^30^.

## Results

### Negative correlations between time to flowering and germination percentage in the F_2_ and BC_1_F_1_ populations

**Figure 1:**
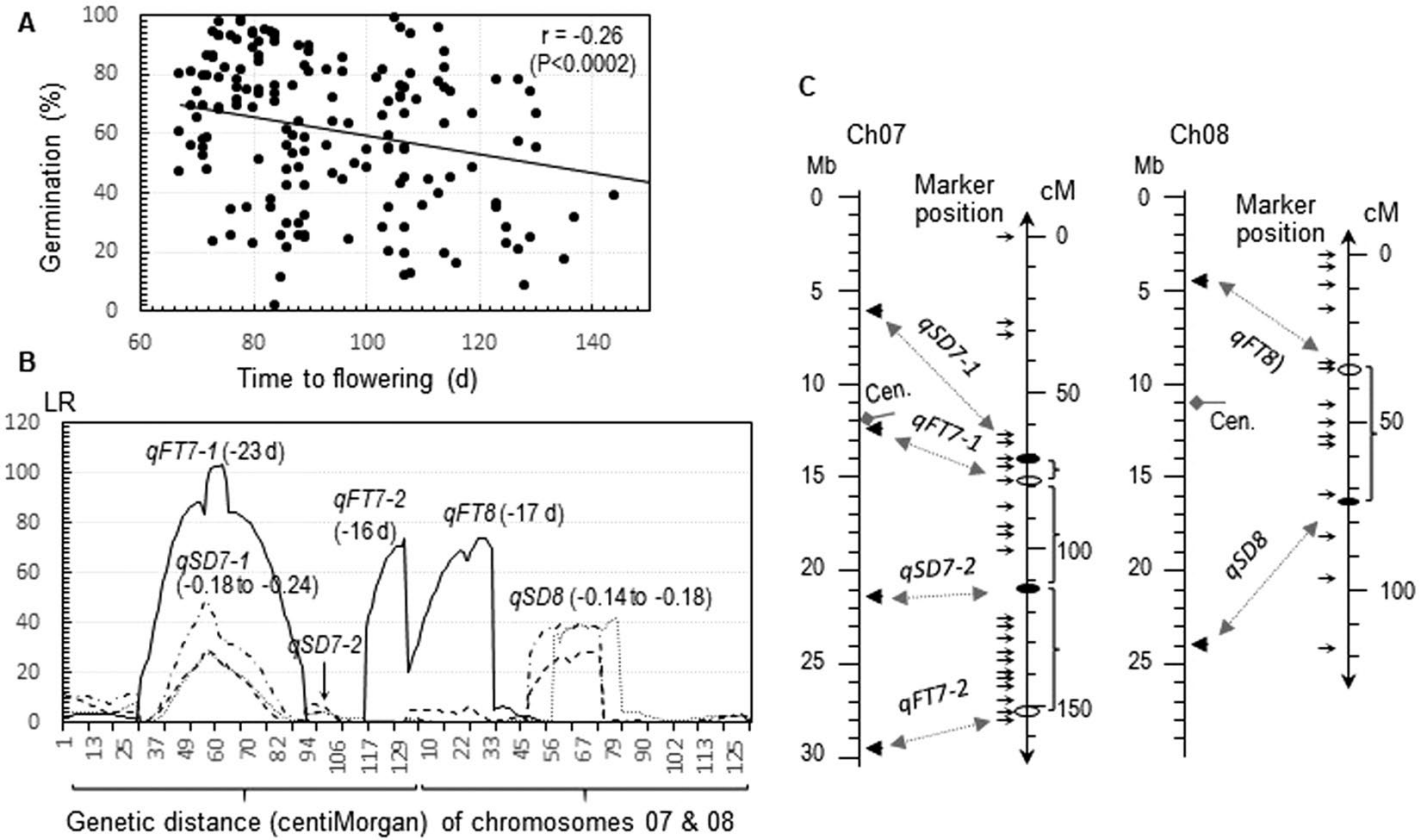
Association and QTL for seed dormancy (SD) and flowering time (FT). (**A**) Scatterplot for the BC_1_F_1_ EM93-1//EM93-1/Ludao population. SD was evaluated by germination percentage of partially after-ripened seeds to estimate the correlation (r). (**B**) Distributions of likelihood ratios (LR) for SD and FT on chromosomes (Ch) 07 and 08. Refer to Supplemental Fig. S2 for a genome-wide scan of the QTL. The negative values in the parentheses indicate that the alleles from Ludao delayed flowering or reduced germination. The arrow indicates the approximate position of *qSD7-2*, which was significant in a BC_2_F_1_ population developed from a BC_1_F_1_ plant^22^. (**C**) Genetic and physical distances between the SD and FT loci on ch 07 and 08. These SD (filled circles) and FT (open circles) loci differentiated between the recurrent parent EM93-1 and the non-recurrent parents Ludao or SS18-2^21,22,32^. The physical positions (mega bases, Mb) of markers and centromere (Cen.) were estimated based on the reference genome sequence^42^.

Late flowering plants tended to have stronger seed dormancy (SD) than early ones in the two BC_1_F_1_ and four F_2_ populations. The time to flowering and the germination percentage of partially after-ripened seeds were negatively correlated (r = −0.24 to −0.45), and the linear correlation accounted for 5–20% of the phenotypic variances in the six populations (Fig. 1A; Supplemental Fig. S1). Heritability for the germinability (0.64–0.95) was relatively high in the populations^20^, suggesting that the negative correlations could be resulted from genes differentiated for the traits.

Photoperiod sensitivity is a major determinant of flowering time (FT) in rice^31^. One of the BC_1_F_1_ populations was multiplied using a split-tiller technique, and the two genetically identical sets of tiller-derived populations were grown in 10- and 14-h day-lengths, respectively, to evaluate the genotypic difference in photoperiod sensitivity of flowering^32^. The correlation strength was similar (r = −0.29) in the two environments (Supplemental Fig. S1A–F), suggesting that seasonal/latitudinal differences in photoperiod may have little influence on the SD-FT association in the short-day plant.

### Linkage disequilibria (LDs) responsible for the negative correlation

The two BC_1_F_1_ populations were analyzed for SD and FT QTL to identify underlying genes for the negative correlations. Eight SD (including *qSD7-1* and 8) and four FT (*qFT3*, *7-1*, *7-2* and *8*) QTL were identified in the BC_1_F_1_ EM93-1//EM93-1/Ludao population (Supplemental Fig. S2). Genetic distance between QTL peaks is 7 cM from *qSD7-1* to *qFT7-1* and about 40 cM from *qSD8* to *qFT8* (Fig. 1B), which are equivalent to 0.07 and 0.33 recombination fractions, respectively. Ludao, a genotype of temperate-ecotype weedy “red” rice, contributed the dormancy-enhancing (germination-reducing) and flowering-delaying alleles to the two sets of linked SD and FT loci. Thus, the close linkage between *qSD7-1* and *qFT7-1* contributed more to the negative correlation than the loose linkage between *qSD8* and *qFT8*.

Six SD (including *qSD7-1*, *7-2* and *8*) and three FT (*qFT7-1*, *7-2* and *8*) QTL were identified in the BC_1_F_1_ EM93-1//EM93-1/SS18-2 populations^23,32^. The loci *qSD7-1*, *qFT7-1*, *qSD7-2* and *qFT7-2* were located on Chr 7 approximately at 7 to 40 cM apart. SS18-2, a genotype of tropical-ecotype weedy “red” rice, contributed the germination-reducing or flowering-delaying alleles to the six loci. Theoretically, all these LD between the adjacent loci on Chrs 7 or 8 could contribute to the negative correlation in the population.

### Positive correlations between time to flowering and germination percentage in advanced progeny lines

**Figure 2:**
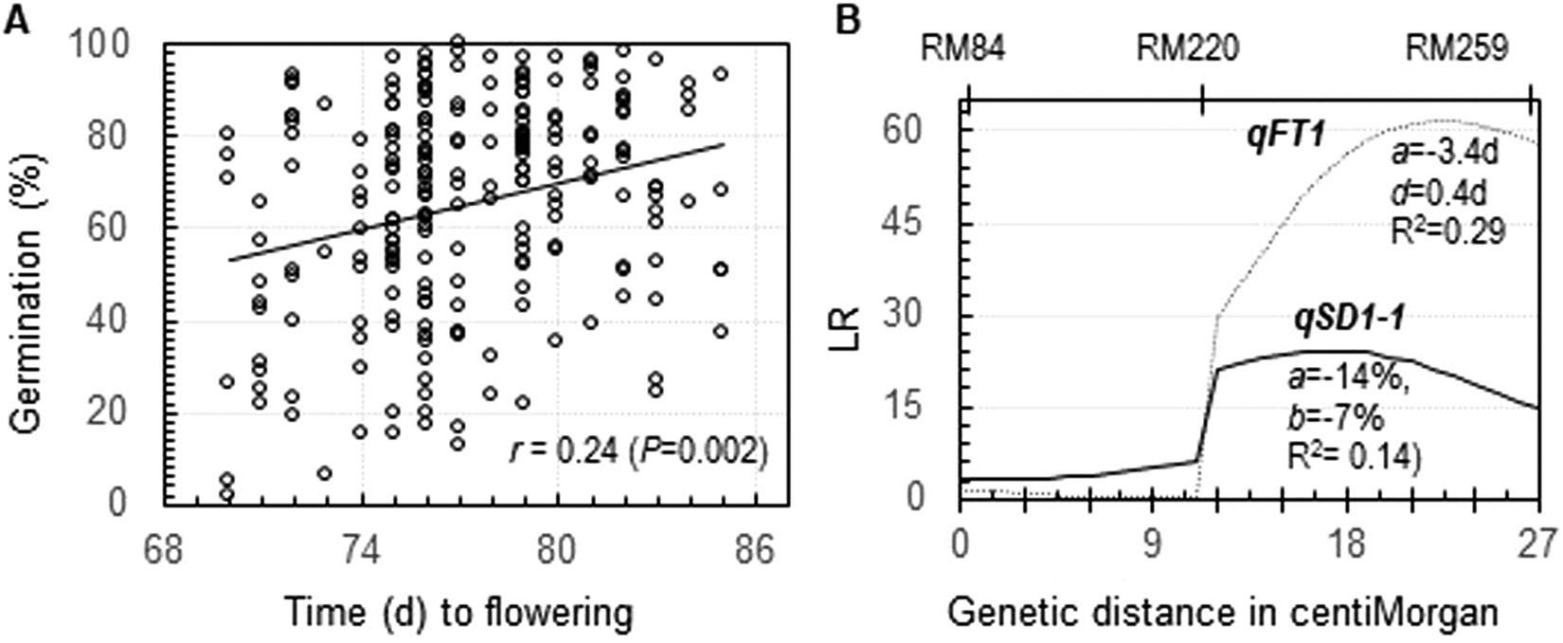
Association and QTL for seed dormancy (SD) and flowering time (FT) in a BC_4_F_3_ population. (**A**) Scatter plot. SD was evaluated by germination percentage to estimate the correlation (r). (**B**) Distributions of likelihood ratios (LR) for SD and FT. This advanced backcross population segregated for three chromosomal segments containing the *qSD1-1*, *7* and *12* loci, respectively^24^. Only the *qSD1-1*-containng segment is shown because the other two segments were not associated with FT. The additive (*a*) and dominance (*d*) effects, and proportion of the variance explained by the QTL (R^2^) were estimated by composite interval mapping. A negative effect indicates that the haplotype from the non-recurrent parent SS18-2 promoted flowering and reduced germination.

Early flowering plants tended to have stronger SD than late flowering plants in advanced generations of progeny lines from two independent families. The family from the BC_4_F_1_ plant contained single copies of the *qSD1-1*, *7-1* and 12 alleles from SS18-2 in the EM93-1 background^24^. A positive correlation (r = 0.24) between time to flowering and germinability was detected in the BC_4_F_3_ population of >200 plants (Fig. 2A). One QTL for FT (*qFT1*) on the *qSD1-1*-containing segment was identified (Fig. 2B). *qSD1-1* and *qFT1* were co-located on a marker interval of <15 cM, with the haplotype from SS18-2 reducing germination and time to flowering. Thus, the linkage between *qSD1-1* and *qFT1* accounted for the positive correlation in the BC_4_F_3_ population.

**Figure 3:**
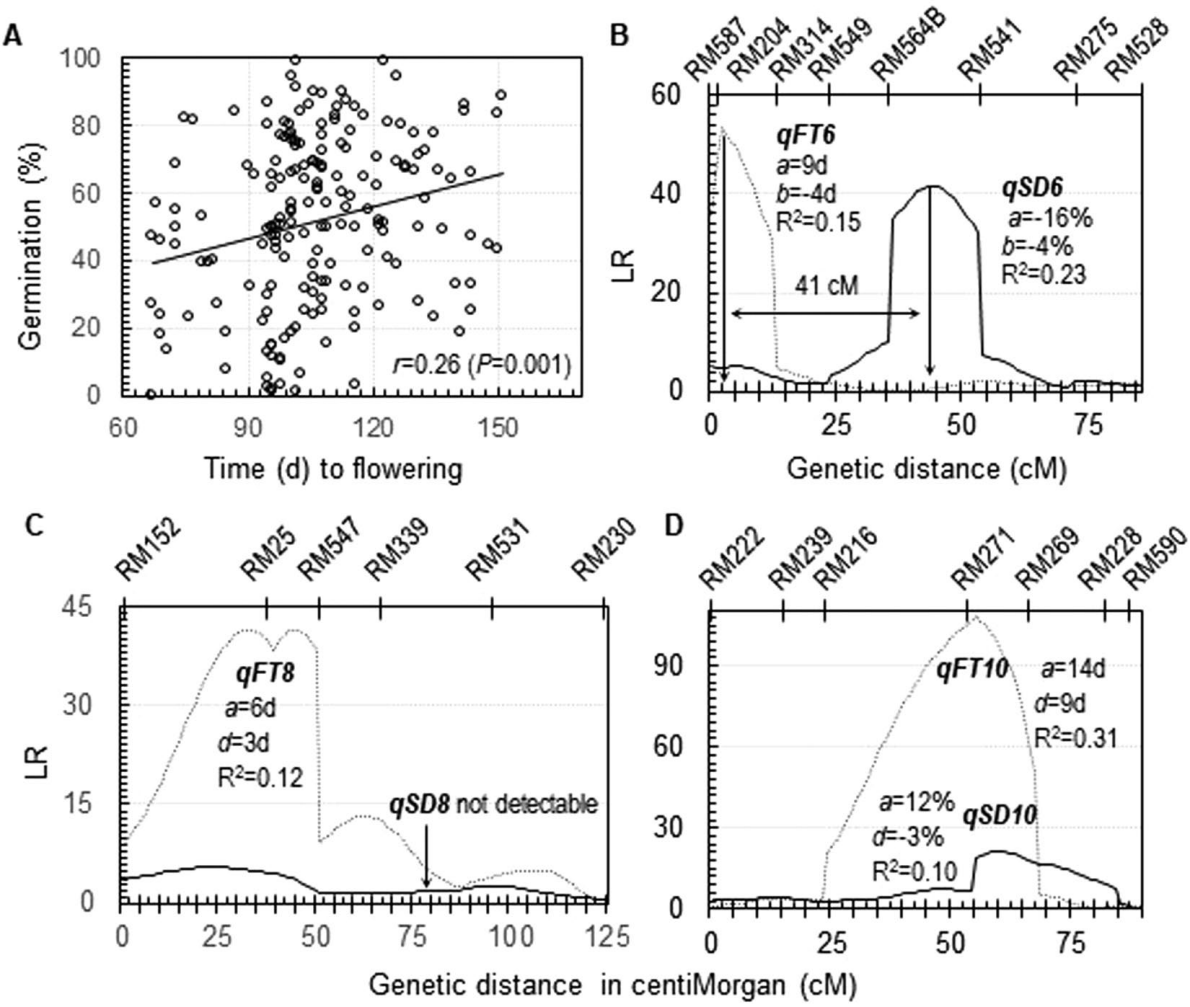
Association and QTL for seed dormancy (SD) and flowering time (FT) in a BC_1_F_2_ population. (**A**) Scatterplot. SD was evaluated by germination percentage to estimate the correlation (r). (**B**–**D**) Distributions of likelihood ratios for QTL mapping. This mapping population was developed from a BC_1_F_1_ plant that contains 10 chromosomal segments from SS18-2 in the EM93-1 background^19^. FT QTL were detected only on chromosomes 06, 08 and 10 (*qFT6*, *8* & *10*). The additive (*a*) and dominance (*d*) effects, and proportion of the variance explained by the QTL (R^2^) were estimated by composite interval mapping. A positive effect indicates that the allele from SS18-2 delayed flowering or promoted germination.

The other family was derived from a BC_1_F_1_ plant, which contained single copies of 10 chromosomal segments from SS18-2 in the EM93-1 background^19^. A similar level of positive correlation (r = 0.26) between time to flowering and germinability was detected in the BC_1_F_2_ population (Fig. 3A). Three QTL for FT (*qFT6*, *8* and *10*) were identified (Fig. 3B–D), including two (*qFT6* and *10*) that were not detected in the BC_1_F_1_ population^32^. Of the three FT loci, *qFT6* is 41 cM from *qSD6*, which may cause a negative correlation, as the haplotype from SS18-2 reduced germination but delayed flowering. *qFT8* did not contribute to the correlation, as *qSD8* was not significant in the BC_1_F_2_ population. Only *qFT10* could cause the positive correlation, as it was co-located with *qSD10*, with the haplotype from EM93-1 reducing germination and time to flowering. Thus, the positive correlation in the BC_1_F_2_ population was an overall effect of a strong LD between *qSD10* and *qFT10* and a weak LD between *qSD6* and *qFT6*.

### A narrowed *qSD1-1/qFT1* haplotype from weedy rice enhanced seed dormancy and promoted flowering

**Figure 4:**
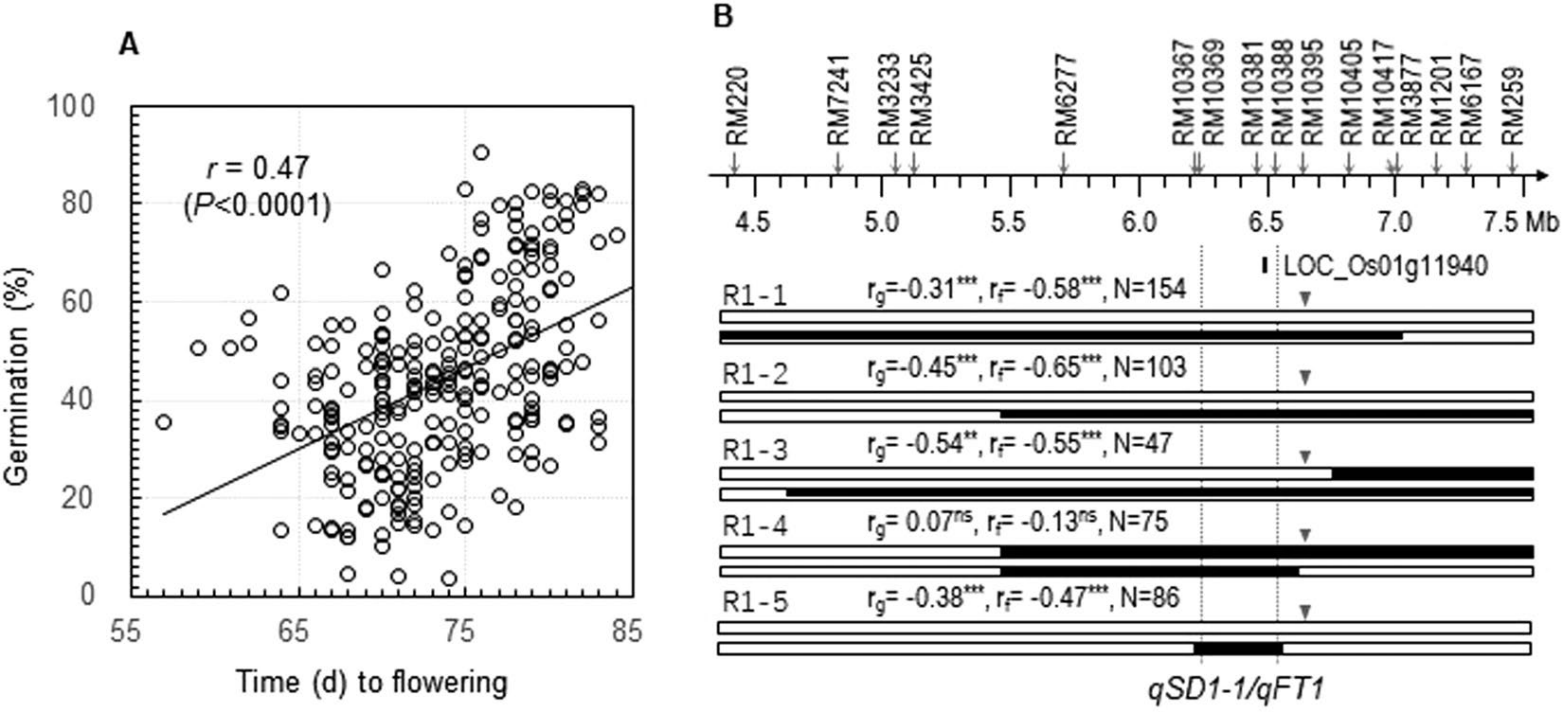
Fine mapping of the *qSD1-1*/*qFT1* cluster. (**A**) Scatterplot for germination percentage and time to flowering time in a BC_4_F_4_ population. This population was derived from a BC_4_F_3_ plant, which was heterozygous only for a *qSD1-1*-containing region (Fig. 2). (**B**) Recombinant genotypes and progeny testing. The recombinants (R#) were selected from the BC_4_F_4_s, delimited with rice microsatellite (RM) markers on the physical map (Top), and represented by chromosomal segments from SS18-2 (dark bars) or EM93-1 (open bars). The arrowheads indicate positons of the markers used to genotype the progeny lines. Correlation coefficients of the marker genotypes with germination percentage (r_g_) or flowering time (r_f_) were estimated based on the N number of plants in a progeny line. The superscripts indicate that the correlations were significant at the probability level of 0.001 (**) or <0.0001 (***), or not significant (ns). The vertical dotted lines delimit a *qSD1-1*/*qFT*-containing region, which encompasses the Os01g11940 locus annotated as a homologous of *Flowering Locus T-Like1* in the reference genome^42^.

The strength of positive correlation between germinability and time to flowering (r = 0.47) was increased in a BC_4_F_4_ population (Fig. 4A). It was developed from a plant in the BC_4_F_3_ population, which was heterozygous for the *qSD1-1/qFT1* interval (Fig. 2B) and had remaining of the genome synchronized by EM93-1. A high-resolution map of 3 mega bases (Mb) was developed for this interval and used to identify recombinants to dissect *qSD1-1* from *qFT1* (Fig. 4B). Significant marker-trait correlations for germination (%; r_g_) and time to flowering (r_f_) were detected in progeny lines from four of the five recombinants, which share a heterozygous interval of <300 kilo bases (Kb) between RM10367 and RM10388 (Fig. 4B). In contrast, there was no correlation in the progeny line from the other recombinant (R1-4) that fixed for the 300-Kb interval with the allele from SS18-2 (Fig. 4B). The co-segregation in the progeny lines indicates that both *qSD1-1* and *qFT1* locate within the 300 Kb.

Genetic analysis for the four progeny lines revealed that pleiotropic effects of *qSD1-1/qFT1* were mainly additive in the isogenic background (Table 1). The *qSD1-1/qFT1* haplotype from SS18-2 inhibited germination and promoted flowering.

**Table 1:** Summary of genetic effects of isolated seed dormancy and flowering time loci in progeny lines derived from selected recombinants.

Recombinants^a^	Progeny lines^b^			
	Germination (%)		Time to flowering (day)	
	*a*	*d*	*a*	*d*
***qSD1-1/qFT1***				
R1-1	−6.9^*^	—	−2.3^***^	—
R1-2	−10.9^***^	—	−3.4^***^	—
R1-3	−12.4^***^	—	−2.6^***^	—
R1-5	−8.9^***^	−7.4^*^	−3.0^***^	—
***qSD10/qFT10***				
R10-1	20.0^***^	8	8.6^***^	—
R10-2	7.6^*^	−15.2^**^	10.1^***^	−6.7^**^
R10-5	10.8^**^	−10.8^*^	13.8^***^	−7.5^**^

^a^Recombinants heterozygous for the narrowed *qSD1-1/qFT1* or *qSD10/qFT10* region (Figs 4 and 5).

^b^Additive (*a*) and dominance (*d*) effects estimated based on Model (1). A positive (negative) value indicates that the allele from the parent line SS18-2 (EM93-1) increased (reduced) germination or the time to flowering at the probability level of 0.05 (*), 0.01 (**) or 0.0001 (***); whereas a dash indicates the effect was not significant in the recombinant-derived progeny lines.

### A narrowed *qSD10/qFT10* haplotype from cultivated rice enhanced seed dormancy and promoted flowering

**Figure 5:**
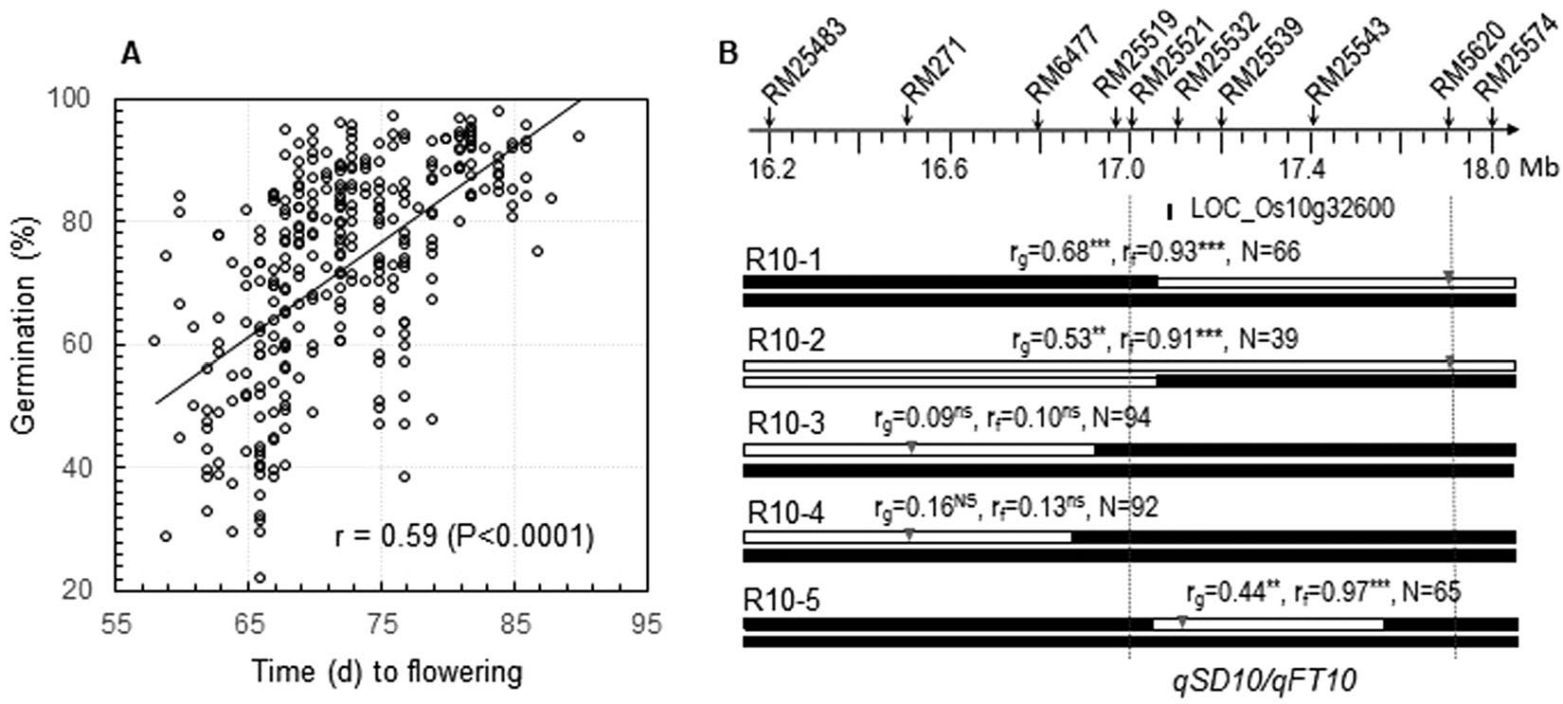
Fine mapping of the *qSD10*/*qFT10* cluster. (**A**) Scatterplot for germination percentage and flowering time and germinability in a BC_1_F_6_ population. This population was advanced from a BC_1_F_2_ plant (Fig. 3) by marker-assisted single-plant selection in each generation to purify the genetic background of *qSD10*. (**B**) Recombinant genotypes and progeny testing. The recombinants (R#) were selected from the BC_1_F_6_ population, delimited with rice microsatellite (RM) markers on the physical map (Top), and represented by chromosomal segments from SS18-2 (dark bars) or EM93-1 (open bars). The arrowheads indicate positons of the markers used to genotype the progeny lines. Correlation coefficients of the marker genotypes with germination percentage (r_g_) or flowering time (r_f_) were estimated based on the N number of plants in a progeny line. The superscripts indicate that the correlations were significant at the probability level of 0.001 (**) or <0.0001 (***), or not significant (ns). The vertical dotted lines delimit a *qSD10*/*qFT10*-containing region, which encompasses the Os10g32600 or *Early heading 1* (*Ehd1*) locus.

The strength of positive correlation between germinability and time to flowering (r = 0.59) was increased in a BC_1_F_6_ population (Fig. 5A). This population was advanced from a BC_1_F_2_ plant for four generations to synchronize the genetic background of *qSD10/qFT10*. A high-resolution map of 2 Mb was developed for the QTL-containing region and used to identify recombinants to dissect *qSD10* from *qFT10* (Fig. 5B). Significant marker-trait correlations were detected in progeny lines from three of the five recombinants, which share a heterozygous interval of <700 Kb between RM25521 and RM5620 (Fig. 5B). In contrast, there was no correlation in progeny lines from the other recombinants (R10-3 and 4) that fixed for the 700-Kb interval with the allele from SS18-2 (Fig. 4B). The co-segregation in the progeny lines indicates that *qSD10* and *qFT10* locate within the 700 Kb.

Genetic analysis for the three progeny lines revealed that *qSD10/qFT10* consisted of both additive and dominance effects on SD and FT in the isogenic background (Table 1). The additive effect of the *qSD10/qFT10* haplotype from EM93-1 inhibited germination and promoted flowering. Compared with *qSD1-1/qFT1*, *qSD10/qFT10* had greater additive effects on SD and FT.

## Discussions

The observed negative and positive correlations between seed dormancy (SD) and flowering time (FT) impart two contrasting patterns of the trait coadaptation in the rice complex. Similar patterns were reported for Arabidopsis (*A. thaliana*), which were related to the latitudinal/altitudinal distribution of temperatures, but not associated with population structures defined by single nucleotide polymorphisms^4,5^. In some other research, the transcript abundance (not genotypes) of *FLOWERING LOCUS C* (*FLC*) was associated with temperature-dependent germinability in Arabidopsis^33^, and the *DELAY OF GERMINATION 1* (*DOG1*) transgene from Arabidopsis delayed flowering in lettuce (*Lactuca sativa*)^34^. Thus, more genetic information is needed to determine if the SD-FT correlations observed in Arabidopsis^4,5^ were resulted from the pleiotropy of *FLC*, *DOG1* or some other genes in the winter annual, long-day plant. Different from Arabidopsis, rice (including weedy relatives) is a short-day plant distributed from tropical to temperate regions. This research provided evidence that the rice complex has a genetic potential to evolve with one of the two contrasting patterns to maximize the plant adaptation to specific ecosystems.

### Late flowering genotypes tend to have stronger SD than early flowering genotypes

This association pattern was observed in >10 experimental populations evaluated in tropical or temperate environments. This pattern may involve one or more sets of LDs on Chrs 1, 3, 6, 7 (4 loci) and 8, with the linkage being closest between *Sdr1* and *HD8* on Chr 3^12^ or closer between *qSD*7*-1* and *qFT7-1* on the pericentromeric area of Chr 7 (Fig. 1C). In addition, the LD between *qSD1-2* and *qFT1* on Chr 1^14^ could be a result of pleiotropy. This is because *qSD1-2* was identified as *semidwarf1* (a gibberellin synthase gene), and its loss-of-function mutants reduced germination and also delayed flowering^35^.

The negative correlation due to LDs may change in strength/direction with populations. A LD decays with generations, resulting in alterations in linkage phase and genetic correlation in descendant populations. QTL analyses identified five haplotypes for the *qSD7-1*, *qFT7-1*, *qSD7-2* and *qFT7-2* loci from six genotypes, including tropical (2) and temperate (2) ecotypes of weedy “red” rice, or *indica* (1) and *japonica* (1) subspecies of cultivated rice (Supplemental Table S1). The six genotypes involve seven recombination events between *qSD7-1* and *qFT7-1* (1), *qFT7-1* and *qSD7-2* (4), or *qSD7-2* and *qFT7-2* (2) (Supplemental Table S1). Additional to the close linkage, natural and artificial selections likely have played opposite roles in maintaining the *qSD7-1/qFT7-1* haplotype. *qSD7-1* is identical to the red pericarp color gene *Rc*, which encodes a transcription factor to activate biosynthesis and accumulation of the dormancy-inducing hormone abscisic acid and the proanthocyanidin pigments in the maternal tissue in red rice^36^. Natural selection favored functional alleles at *qSD7-1/Rc* for enhanced adaption in wild/weedy red rice^37^, which could also increase the frequency of a late-flowering allele at *qFT7-1*, due to the close linkage. Whereas, artificial selection favored the white pericarp-colored mutants^38^, which were the recombinant between *qSD7-1* and *qFT7-1* in some cultivars, such as Nipponbare (Supplemental Table S1). The recombination could alter the direction of the correlation associated with the *qSD7-1/qFT7-1* haplotype.

Factors influencing the negative correlation also include epistatic or genotype-by-environment (G-by-E) interactions of the underlying genes. Our previous research demonstrated that the *FT7-1*, *7-2* and *8* loci interacted with each other and also with photoperiods to regulate the phenotypic variation in FT^33^. Similarly, both *SD1-1* and *7-1* were involved in the G-by-E (temperature) interaction, as the loci had a greater effect on germination inhibition in low (mean 21 °C) than in high (mean 27 °C) temperatures during seed development^24^. Depending on genes, these complex interactions could influence a negative or a positive correlation.

### Early flowering genotypes tend to have stronger SD than late flowering genotypes

This association pattern and genetic basis were first described in this research. Two independent sets of co-located SD/FT loci, *qSD1-1/qFT1* and *qSD10/qFT10*, are responsible for the positive correlation. These underlying genes are different from those for the negative correlations in several aspects. Firstly, the two sets of QTL were all segregating in the BC_1_F_1_ population, but none of them was detectable in the early generation in which the overall correlation was negative. Thus, the negative *vs*. the positive pattern is similar to the dominant *vs*. the recessive allele at a locus. Secondly, the *qSD1-1/qFT1* and *qSD10/qFT10* haplotypes that enhance dormancy and promote flowering distribute in weedy (SS18-2) and cultivated (EM93-1) rice, respectively. It is likely that haplotype variants of the two sets originated from different ecotypes. And lastly, both *qSD1-1/qFT1* and *qSD10/qFT10* were isolated as single Mendelian factors. The co-segregation across several generations suggests that the positive pattern may arise from pleiotropy.

### Synergy and selective advantage of the SD-FT associations

**Figure 6:**
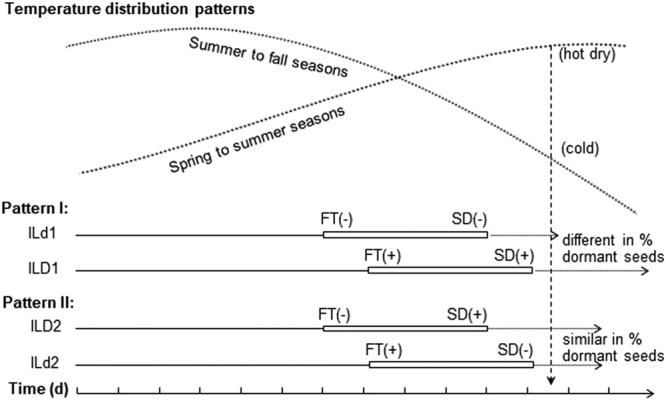
Modeling synergetic effects of associations between seed dormancy (SD) and flowering time (FT). Isogenic lines (IL) are used to depict two association patterns. Pattern I, the dormant [SD(+)] genotype (ILD1) takes a longer time to flower [FT(+)] than the non-dormant [SD(−)] genotype (ILd1); Pattern II, the dormant genotype (ILD2) takes a shorter time to flower [FT(−)] than the non-dormant genotype (ILd2). Assumed that ILs germinate at 0 d, are similar in length for flowering to maturation (open bars), and start the dormancy release, or after-ripening (AR), immediately after maturation. The degree of primary dormancy and the AR time required for an IL to release the dormancy (arrow-headed dotted lines) are determined by synergetic effects of the dormancy gene and the environmental factors (mainly the temperatures simulated by dotted curve lines) predetermined by the flowering time. The vertical dash line indicates a time point, when environmental temperatures change from a level good for AR to the level favorable for the dormancy maintenance or the secondary dormancy induction. At the hypothesized time point, genotypic difference in the percentage of dormant seeds entering into the soil seed bank would be greater for Pattern I than for Pattern II.

Synergy of an SD-FT association would include interactional effects on the dormancy development and release (after-ripening, AR) in an environment where the plant growth duration is critical for seed survival or germination. For a locally adapted population, FT determines the maturation time, as the period from flowering to maturation is relatively constant; and FT also determines environmental conditions for seed development to a large extent, and to some extent for the AR time required for dormancy release. Thus, for a negative SD-FT association in an isogenic background, the late-flowering dormant genotype would have a higher percentage of dormant seeds than the early flowering non-dormant genotype at a given time point after maturation or dispersal, such as Pattern I in Fig. 6. However, for a positive SD-FT association, the early-flowering dormant and the late-flowering non-dormant genotype would be similar for the percentage of dormant seeds at the time, such as Pattern II in Fig. 6. The modeling results suggest that the two contrasting patterns could be different in the proportion of dormant seeds left in the field at a time point, when the temperature gradually changes from a level favorable for the dormancy release to a level good for the dormancy maintenance or the induction of secondary dormancy (Fig. 6). At this critical time, the predicted genotypic difference in the dormancy or germination level is greater for Pattern I than for Pattern II. In such an environment, a natural selection could cause a larger deviation of genotypic frequency from a genetic equilibrium for Pattern I than for Pattern II in the germinable or non-germinable subpopulation.

Temperature during seed development is a major factor influencing the selective advantage of a SD-FT association. In Arabidopsis, plants setting seeds at ≤14 °C had stronger dormancy than those at >14 °C^39^. The association pattern in Arabidopsis is similar to the positive correlation pattern in rice, as the early flowering plants could experience relatively low temperatures to set dormant seeds than the late ones. In the rice complex, the late-flowering, dormant genotype could have a selective advantage over the early flowering, weakly-dormant genotype to survive in hot dry, or cold, seasons. This is supported by: (1) the “dominance” of the negative over the positive pattern in the F_2_ and BC_1_F_1_ populations; (2) the greater synergetic effect for Pattern I than for Pattern II (Fig. 6); and (3) the divergence of SD genes in germination response to seed development temperatures^24^. In shepherd’s purse, it was postulated that early-flowering weakly dormant genotypes might have a selective advantage over late-flowering dormant genotypes to maintain heteromorphic seeds in arable areas^3^.

A weed population often mimics accompanying cultivars for agronomic characters, such as early flowering, to avoid harvesting^40^. Thus, the selection pressure imposed by some agricultural practices may increase the frequency of the early-flowering dormant genotype, such as the line ILD2 in Pattern II (Fig. 6). This explains why the “recessive” pattern of positive correlation could be maintained in populations of weedy rice. Therefore, the synergy of a SD-FT association varies with ecosystems, and its effect on plant adaptation cannot be overstated, as the observed correlations accounted for only a small proportion of the phenotypic variation in this and the other research.

### Breeding application of the *qSD1-1/qFT1* and *qSD10/qFT10* haplotypes

The narrowed *qSD1-1/qFT1* and *qSD10/qFT10* regions contain the predicted loci Os01g11940 and Os10g32600, respectively (Figs 4B and 5B). Os01g11940 is homologous to the flowering-promoting gene *Flowering Locus T* (*FT*) in Arabidopsis^41^. Os10g32600 is annotated as a Myb family transcript factor (TF) gene^42^ and was identified as the *Early Heading date 1* (*Ehd1*) QTL^43,44^. Thus, the *FT* homologue and *Ehd1* are the best candidates for *qFT1-1* and *10*, respectively. Both *FT* and *Ehd1* were not reported to have an effect on SD or germination in Arabidopsis or rice. Research is being conducted to clone and characterize the QTL underlying genes.

Selection in crop breeding for growth duration may influence germinability, and *vice versa*, because of the SD-FT associations. Most cultivars of rice have an insufficient degree of SD to overcome the pre-harvest sprouting (PHS) problem^45^. PHS could lower germinability (<70%) in seed production of hybrid rice. In addition to weak SD, parental lines of hybrid rice were selected from early flowering genotypes to reduce the hybrid vigor for growth duration, and applied with gibberellic acid (GA) to promote panicle elongation. The GA application also induces germination on the plants and consequently lowers seed storability and germinability. Many QTL reported for PHS remain to be confirmed or determined for association with SD or FT^46–49^. Thus, the isolated *qSD1-1/qFT1* and *qSD10/qFT10* haplotypes would be idea candidates to develop PHS-resistant, early-maturation varieties.

## Electronic supplementary material

Supplementary information
