# Supplementary material for: Two Contrasting Patterns and Underlying Genes for Coadaptation of Seed Dormancy and Flowering Time in Rice
Source: Sci Rep. 2018 Nov 14;8:16813. doi: 10.1038/s41598-018-34850-5 (PMC6235893; doi:10.1038/s41598-018-34850-5)
Supplement: Supplementary file 1 — Supplementary information [file 41598_2018_34850_MOESM1_ESM.pdf]

Two Contrasting Patterns and Underlying Genes for Coadaptation of Seed Dormancy and Flowering Time in Rice

Xing-You Gu[1], Wirat Pipatpongpinyo[1], Lihua Zhang[1], Yuliang Zhou[2], Heng Ye[1], Jiuhuan Feng[1]

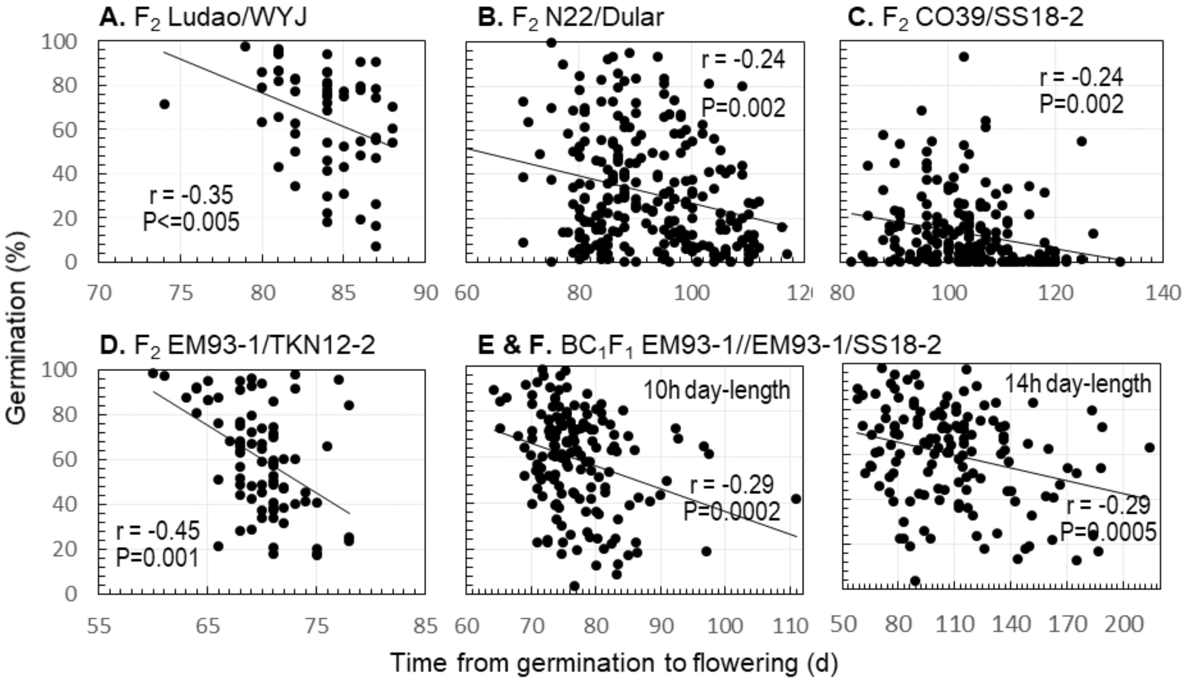

Supplemental Figure S1. Scatter plots showing negative correlations between germination percentage and time to flowering in primary segregating populations. **A-D**. F_2 populations. These populations were evaluated for heritability of seed dormancy[20]. **E-F.** Two genetically identical BC_1F_1 populations. The population was multiplied by a split-tiller technique at the seedling stage and the tiller-derived populations were grown in 10- and 14-h day-length environments, respectively[21,32]. Ludao, SS18-2 and TKN12-2 are pure lines of weedy red rice; and the remaining parents are *indica*- (CO39, Dular, EM93-1 and N22) or japonica- (WYJ) types of cultivated rice.

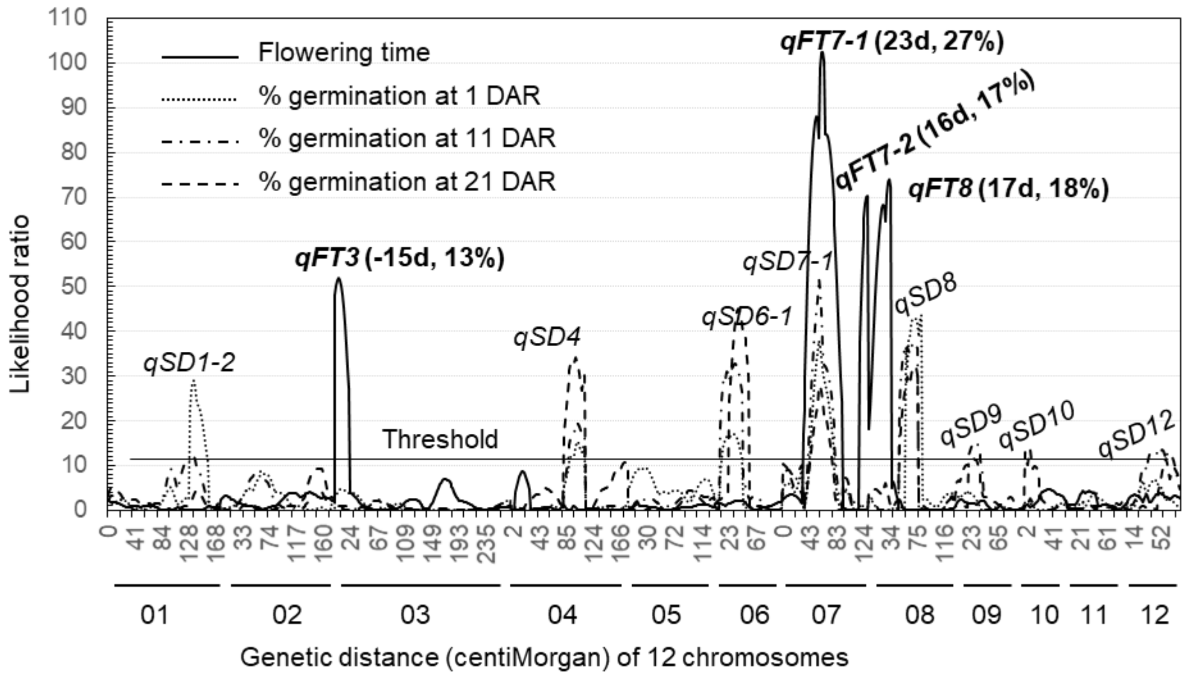

Supplemental Figure S2. Genome-wide scan for QTL associated with flowering time (qFT) and seed dormancy (qSD) in the BC_1F_1 EM93-1//EM93-1/LD) population. Data in the parentheses after a qFT are the genetic effect on time (d) to flowering, with a positive value indicating that the allele from LD delays flowering, and the proportion of variance explained by the QTL. Seed dormancy was evaluated by percentage germination at 1, 11 and 12 days of after-ripening (DAR)[22].

Supplemental Table S1. Haplotypic variants for four loci on chromosome 7 in parental lines of weedy or cultivated rice used to map the seed dormancy (qSD) and flowering time (qFT) loci.

Line[a]	*qSD7-1*[b]	*qFT7-1*	*qSD7-2*	*qFT7-2*	Type (pericarp color; origin)
SS18-2	+	+	+	+	Weedy (red; Thailand)
Ludao	+	+	-	+	Weedy (red; China)
US1	+	+	-	-	Weedy (red; USA)
TKN12-2	+	+	-	-	Weedy (red; Nepal)
Nipponbare	-	+	-	+	Cultivated *japonica* (white; Japan)
EM93-1	-	-	-	-	Cultivated *indica* (white; breeding line)
Recomb.[c]	1	4	2		Total 7

[a] EM93-1 was crossed with SS18-2[21,32], US1[14], Ludao[22], TKN12-2 and Nipponbare (Gu, unpublished data) to map the quantitative trait loci. Haplotypes for the four loci are represented by alleles that increase (+), or reduce (-), seed dormancy and time to flowering.

[b] This locus is identical to the red pericarp color gene (*Rc*) in weedy red rice[36].

[c] Number of recombinants between adjacent loci, based on the wild-type (SS18-2) and mutant (EM93-1) controls.
